# Ontogenetic changes to metacarpal trabecular bone structure in mountain and western lowland gorillas

**DOI:** 10.1111/joa.13630

**Published:** 2022-02-04

**Authors:** Kim Deckers, Zewdi J. Tsegai, Matthew M. Skinner, Angel Zeininger, Tracy L. Kivell

**Affiliations:** ^1^ Skeletal Biology Research Centre, School of Anthropology and Conservation University of Kent Canterbury, Kent UK; ^2^ Department of Human Evolution Max Planck Institute for Evolutionary Anthropology Leipzig Germany; ^3^ Department of Evolutionary Anthropology Duke University Durham North Carolina USA

**Keywords:** African ape, cancellous bone, development, functional morphology, hand, knuckle‐walking

## Abstract

The trabecular bone morphology of adult extant primates has been shown to reflect mechanical loading related to locomotion. However, ontogenetic studies of humans and other mammals suggest an adaptive lag between trabecular bone response and current mechanical loading patterns that could result in adult trabecular bone morphology reflecting juvenile behaviours. This study investigates ontogenetic changes in the trabecular bone structure of the third metacarpal of mountain gorillas (*Gorilla beringei beringei*; *n* = 26) and western lowland gorillas (*Gorilla gorilla gorilla*; *n* = 26) and its relationship to expected changes in locomotor loading patterns. Results show that trabecular bone reflects predicted mechanical loading throughout ontogeny. Bone volume fraction, trabecular thickness and trabecular number are low at birth and increase with age, although degree of anisotropy remains relatively stable throughout ontogeny. A high concentration of bone volume fraction can be observed in the distopalmar region of the third metacarpal epiphysis in early ontogeny, consistent with the high frequency of climbing, suspensory and other grasping behaviours in young gorillas. High trabecular bone concentration increases dorsally in the epiphysis during the juvenile period as terrestrial knuckle‐walking becomes the primary form of locomotion. However, fusion of the epiphysis does not take place until 10–11 years of age, and overall trabecular structure does not fully reflect the adult pattern until 12 years of age, indicating a lag between adult‐like behaviours and adult‐like trabecular morphology. We found minimal differences in trabecular ontogeny between mountain and western lowland gorillas, despite presumed variation in the frequencies of arboreal locomotor behaviours. Altogether, ontogenetic changes in *Gorilla* metacarpal trabecular structure reflect overall genus‐level changes in locomotor behaviours throughout development, but with some ontogenetic lag that should be considered when drawing functional conclusions from bone structure in extant or fossil adolescent specimens.

## Graphical abstract

This study investigates the ontogenetic changes in trabecular structure in western lowland and mountain gorilla third metacarpals and how these changes may reflect locomotor behaviour throughout development. We found that metacarpal trabecular structure reflects a higher frequency of grasping in early ontogeny and a shift to predominant knuckle walking locomotion in later ontogeny, but with an 'adaptive lag' between adult‐like behaviour and adult‐like trabecular structure that should be considered when analysing juvenile or young adult fossil specimens.

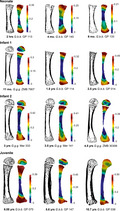

## 1 INTRODUCTION

Reconstruction of locomotor behaviour in fossil hominins can only be done by inferring behaviour from skeletal features. However, behavioural reconstructions based on external bone morphology often lead to conflicting conclusions, especially with regards to the degree of arboreality of fossil hominin species (e.g., Dominguez‐Rodrigo et al., 2013; Haile‐Selassie et al., 2012; Ruff et al., 2016; Stern & Susman, 1983; Wallace et al., 2020). Internal bone structure may be useful in inferring the behaviour of extinct hominins as it has been shown to (re‐)model in response to the magnitude and direction of mechanical loading during life (Barak et al., 2011; Biewener et al., 1996; Cowin, 2002; Currey, 2002; Pontzer et al., 2006). Variation in trabecular bone morphology has been successfully correlated with differences in locomotor behaviours within certain extant primates and skeletal elements (e.g., Dunmore et al., 2019; MacLatchy & Müller, 2002; Maga et al., 2006; Matarazzo, 2015; Ryan & Ketcham, 2002; Tsegai et al., 2013), but has been less successful in other primate species, especially in the upper limb (e.g., Ryan & Walker, 2010; Scherf et al., 2013; Schilling et al., 2014).

One explanation for why functional signals within trabecular bone vary across taxa or skeletal elements may be that adult internal bone structure is reflecting behaviours practised throughout ontogeny (Pearson & Lieberman, 2004), thus creating a lag in the response to mechanical loading. This lag has been observed in the trabecular bone morphology of pigs (Tanck et al., 2001), showing that bone volume fraction and stiffness increased rapidly from 6 weeks of age, while the degree of anisotropy did not change until after 23 weeks. Within the human calcaneus, ontogenetic changes broadly reflect maturity of bipedal gait, but the adult‐like pattern within different regions of the calcaneus is attained through distinct developmental trajectories (Saers et al., 2020). Moreover, variation in trabecular structure within the adult human calcaneus has been shown to correlate with activity levels during childhood and adolescence, rather than during adulthood (Pettersson et al., 2010). However, the influence of ontogenetic activity on internal bone structure has not been thoroughly studied for non‐human primates (Ragni, 2020; Ruff et al., 2013, 2018; Tsegai et al., 2018). This study aims to help fill this knowledge gap by investigating changes in trabecular structure throughout ontogeny within the gorilla third metacarpal. We explore the degree to which trabecular bone modelling (sensu Barak, 2020) is driven by changes in behaviour throughout development in two *Gorilla* species—western lowland gorillas (*Gorilla gorilla gorilla*) and mountain gorillas (*Gorilla beringei beringei)*—that differ in their locomotor patterns.

### 1.1 Ontogeny of *Gorilla* locomotor behaviour

The primary form of locomotion for all adult *Gorilla* species is terrestrial knuckle‐walking (Doran, 1996, 1997; Remis, 1995, 1998) but there is substantial variation in the degree of arboreality among individuals both within and between species. Detailed data on how locomotion and activity patterns change throughout ontogeny have only been reported for mountain gorillas (*G. b. beringei*) in the Virunga Mountains (Parc National des Volcans; Doran, 1997) and thus what is described below is primarily based on this population only. However, the general pattern of ontogenetic change is thought to be similar for western lowland gorillas (*G. g. gorilla*) until approximately 4 years of age, when terrestrial knuckle‐walking becomes the most frequent form of locomotion in both species (Remis, 1995) and here we assume the same.

**FIGURE 1:**
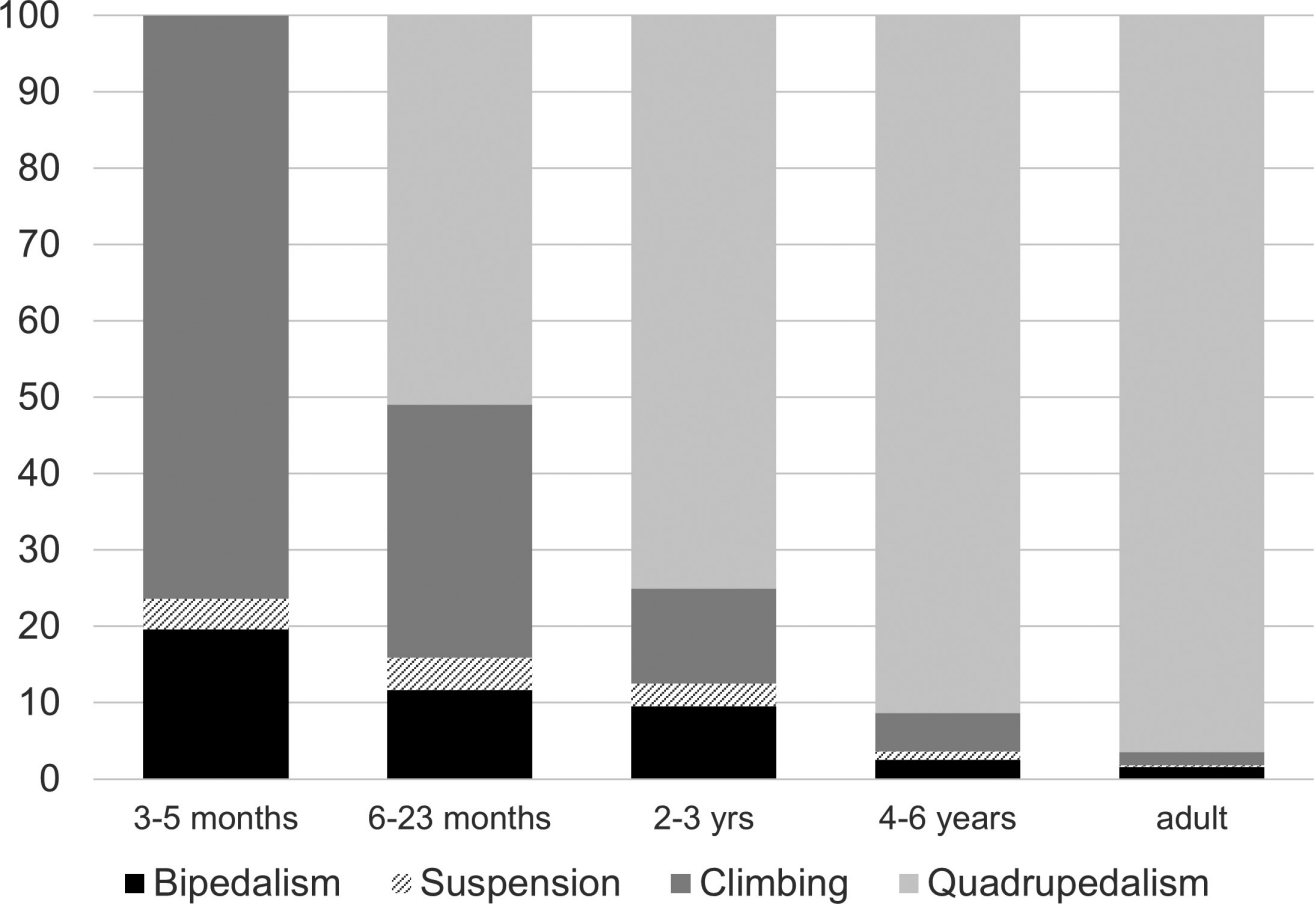
Changes in *Gorilla beringei beringei* locomotor activity throughout ontogeny represented as frequency relative to total locomotor time. Data modified from Doran (1997)

Gorillas spend the first 3 months of their life clinging to their mother and thus do not engage in independent locomotor behaviour (Doran, 1997; Figure 1). Between approximately 6 and 10 months of age, locomotor activity increases significantly with nearly 60% of all locomotion occurring in the trees, and 39% of this being primarily palmigrade quadrupedal locomotion (Doran, 1997). Between 10 and 15 months of age, knuckle‐walking becomes increasingly more frequent (and palmigrady less frequent) and arboreal behaviours (suspension and vertical climbing) become more frequent as well. Suspension and vertical climbing increases until approximately 2 years of age, when knuckle‐walking then becomes the primary form of quadrupedal locomotion and climbing behaviours start to decrease (though still much higher than in adults). Doran (1997) reports that by 4 years of age, the frequency of terrestrial knuckle‐walking is similar to that of adults (85% of all locomotion), but that the rate of locomotor activity is much higher and then decreases with age. Adult mountain gorillas spend at least 90% of their time practicing terrestrial knuckle‐walking, and up to 98% in male adults in particular (Doran, 1997; Figure 1).

In contrast to mountain gorillas, adult western lowland gorillas have been reported to spend at least 20% of their time engaging in arboreal behaviours (Remis, 1995, 1998). No studies of which we are aware have documented the ontogenetic changes in locomotion in western lowland gorillas, so it is unclear if such differences in the frequency of arboreality also occur throughout development. Moreover, there will likely be differences in the frequency of locomotor behaviours across different populations of lowland and mountain gorillas that live in varied ecological settings, which have yet to be formally documented. However, it is possible that the increased frequency of arboreal behaviours in western lowland gorillas, especially when consistently practiced throughout adulthood, will be reflected in the internal bone structure.

### 1.2 Metacarpal internal bone structure

Development of the metacarpus in utero and after birth has only been extensively studied in humans (Scheuer et al., 2000), but the general developmental trajectory is assumed to be similar in African apes (Bolter & Zihlman, 2003; Kerley, 1966). A single primary ossification centre for each of the metacarpals 2–5 develops in utero and is located distally (Rolian, 2016). During ontogeny, cartilage cells are deposited at the growth plate and subsequently ossify, which results in primary trabecular bone being laid down just underneath the growth plate (Scheuer et al., 2000). The secondary ossification centre for the metacarpal, located in the epiphysis, develops during the first few years after birth. As the bone matures and ossifies, primary trabecular bone is remodelled into secondary trabecular bone under the influence of systemic and mechanical loading. The third metacarpal epiphysis is fully fused in humans between 14–16 years of age (Scheuer et al., 2000) and in chimpanzees by 9 years of age (Kerley, 1966), but has not been formally studied in gorillas.

Within the primate hand skeleton, the internal bone structure of the third metacarpal has been comparatively well‐studied (e.g. Barak et al., 2017; Chirchir et al., 2017; Dunmore et al., 2019; Dunmore, Bardo, et al., 2020; Matarazzo, 2015; Tsegai et al., 2013; Zeininger et al., 2011). The third metacarpal’s central position within the hand means that it regularly incurs load during most locomotor and manipulative activities. A study of pressure distribution during terrestrial knuckle‐walking in captive lowland gorillas showed that peak pressures were highest on the third digit (Matarazzo, 2013). During knuckle‐walking, the metacarpophalangeal joint is hyperextended (Matarazzo, 2013; Susman, 1979; Tuttle, 1969), while during arboreal or manipulative grasping, the metacarpophalangeal joint is typically flexed to varying degrees depending on the size of the substrate or object (Neufuss et al., 2017; Sarmiento, 1994).

The trabecular structure of the third metacarpal has been shown to be consistent with the presumed joint postures and loads incurred during the most frequent hand use behaviours in adult hominoids. African apes show an increase in trabecular bone volume in both the dorsal and palmar aspects of the metacarpal heads, consistent with the metacarpophalangeal joint position during both knuckle‐walking and arboreal grasping (Chirchir et al., 2017; Dunmore et al., 2019; Matarazzo, 2015: Tsegai et al., 2013, but see Barak et al., 2017). The African ape trabecular pattern is distinct from the distopalmarly‐concentrated trabecular bone volume in orangutan metacarpal heads and asymmetrical patterning of human metacarpals (Chirchir et al., 2017; Dunmore, Bardo, et al., 2020; Dunmore, Skinner, et al., 2020; Stephens et al., 2018; Tsegai et al., 2013).

However, studies of the ontogeny of trabecular bone in primates have primarily focused on humans and on the lower limb. For example, ontogenetic changes in trabecular architecture within the tibia, femur and calcaneus have been linked to the onset and maturity of bipedal walking (Gosman & Ketcham, 2009; Raichlen et al., 2015; Ryan & Krovitz, 2006; Saers et al., 2020). Generally, bones of the human lower limb follow a similar pattern of trabecular change: at birth they have the highest bone volume fraction (BV/TV) and connectivity than at any other stage of life, followed by a dramatic reduction in both parameters around 1 year of age, and finally a more gradual increase in BV/TV, connectivity and trabecular alignment between 2 and 4 years of age as independent bipedalism develops and gait matures (Gosman & Ketcham, 2009; Milovanovic et al., 2017; Ryan & Krovitz, 2006; Saers et al., 2020). This pattern of an initial reduction in trabecular robusticity until around 1 year of age is also found in the human humerus, suggesting a potential systemic pattern of trabecular ontogeny across the modern human (and Neandertal) skeleton (Chevalier et al., 2021; Ryan et al., 2017). However, it has also been shown that the distinctive trabecular pattern of the human pelvis is largely present at birth and perhaps not greatly influenced by mechanical loading (Abel & Macho, 2011; Cunningham & Black, 2009).

Our understanding of ontogenetic trabecular bone changes in non‐human apes is much more limited. Currently the ontogeny of trabecular structure has only been studied in the African ape calcaneus (Zeininger, 2013), third metacarpal base and capitate (Ragni, 2020) and the chimpanzee humerus, femur and tibia (Tsegai et al., 2018). These studies suggest that changes in trabecular morphology may be consistent with locomotor transitions (Tsegai et al., 2018). However, these studies also suggest that at least these skeletal elements follow a different ontogenetic pattern than that of modern humans. For example, the dip in BV/TV during infancy found in humans is not seen in the African ape calcaneus (Zeininger, 2013) or long bones (Tsegai et al., 2018). In contrast, humans and chimpanzees share a pattern of increasing trabecular thickness during ontogeny (Chevalier et al., 2021; Gosman & Ketcham, 2009; Ryan et al., 2017; Saers et al., 2020; Tsegai et al., 2018), perhaps as the trabecular structure adjusts to increasing body size during growth. Moreover, Tsegai et al. (2018) found a decrease in degree of anisotropy within the humerus and tibia during the first 5 years of life, while anisotropy remained relatively constant in the femur, suggesting different bones may respond to mechanical loading in different ways throughout ontogeny.

To our knowledge, only one study has focused on ontogenetic changes in trabeculae within the metacarpals of great apes. Ragni (2020) investigated the proximal third metacarpal (and capitate) of chimpanzees and gorillas and found that anisotropy remained constant while BV/TV decreased throughout ontogeny. This result is in contrast to the increase in BV/TV in the chimpanzee femur and tibia and changes in anisotropy observed in chimpanzee humerus and tibia (Tsegai et al., 2018). Furthermore, Ragni (2020) found no significant differences in the ontogenetic changes to trabecular bone between chimpanzees and gorillas despite a higher frequency of arboreal behaviours in the former. However, this study analysed trabeculae using a volume‐of‐interest approach in the base of the third metacarpal and capitate, a relatively immobile joint compared to the metacarpophalangeal joint. Given the strong locomotor signals found in the trabecular structure of adult hominoid third metacarpal heads (Chirchir et al., 2017; Dunmore et al., 2019; Matarazzo, 2015: Tsegai et al., 2013), investigation of ontogenetic changes throughout this bone, including changes at the growth plate and epiphysis (where present) may reveal more clear functional signals consistent with locomotor changes during growth.

This study investigates trabecular ontogeny within the third metacarpal of both western lowland and mountain gorillas using a whole‐bone approach to test whether changes in trabecular structure reflect changes in the frequency of locomotor behaviour throughout development. We predict that:

1. Changes in trabecular bone morphology during ontogeny will follow the same pattern as that observed in the whole‐region analysis of the upper limb of chimpanzees (Tsegai et al., 2018), such that BV/TV will initially be low and increase throughout ontogeny. We expect the degree of anisotropy (DA) to remain relatively constant throughout life in the base, as observed by Ragni (2020), and for anisotropy in the epiphysis to be initially isotropic and become more anisotropic as the variation in locomotor behaviours decreases throughout ontogeny.

If changes in locomotor behaviour through ontogeny are reflected in the trabecular structure, we predict that:

- BV/TV will be greater in the palmar region of the metacarpal epiphysis during early ontogeny, reflecting flexed‐finger grasping during clinging and arboreal behaviours. As knuckle‐walking becomes more frequent, we predict BV/TV to be greater in the dorsal region of the metacarpal epiphysis, reflecting a hyperextended metacarpophalangeal joint posture.
- This shift from palmar to dorsal concentration of BV/TV will also be reflected in the metaphysis. However, we expect any changes in trabecular structure of the metacarpal base related to locomotor behaviour to be less pronounced than those of the epiphysis given limited mobility at the carpometacarpal joint.
- Trabecular structure will begin to differ between species around 4 years of age when mountain gorillas shift from a mixture of arboreal and terrestrial locomotor behaviours to mainly terrestrial knuckle‐walking (Doran, 1997), while western lowland gorillas are thought to maintain a higher frequency of arboreal behaviour throughout life (Remis, 1995; Ruff et al., 2013). This shift will be reflected in relatively greater BV/TV and DA in the dorsal region of the mountain gorilla metacarpal epiphysis compared with western lowland gorillas.

## 2 MATERIALS AND METHODS

### 2.1 Study sample

The study sample consists of immature and adult third metacarpal specimens from 26 *G. g. gorilla* and 26 *G. b. beringei* individuals (Table 1). Western lowland gorilla specimens are curated at the Powell‐Cotton Museum (PC), UK and the Natural History Museum Berlin—Leibniz Institute for Evolution and Biodiversity Science (ZMB), Germany. The mountain gorilla sample comprises individuals from the Mountain Gorilla Skeletal Project (MGSP), which contains Virunga mountain gorilla specimens collected from Volcanoes National Park, Rwanda and are curated at the Dian Fossey Gorilla Fund International’s Karisoke Research Centre in Rwanda in partnership with the Rwanda Development Board. All *G. g. gorilla* and *G. b. beringei* individuals were wild and specimens did not show any signs of pathology. Trabecular bone morphology was analysed in the base, the distal metaphysis (i.e. beneath the growth plate), and the epiphysis of the third metacarpal. In early ontogeny, the epiphysis is unfused and highly cartilaginous and was therefore not present for every individual in the sample.

**TABLE 1:** Age and species distribution of the *Gorilla* study sample. ‘M', male; ‘F', female; ‘U', unknown; ‘no.’, number

Age category	Age	No. individuals (M/F/U)		No. per bone section			Resolution
		*Gorilla gorilla gorilla*	*Gorilla beringei beringei*	Base	Metaphysis	Head	
Neonate	0–6 months	0/1/0	1/2/0	4	4	0	0.019–0.029
Infant 1	7–24 months	2/3/0	3/2/0	9	9	1	0.024–0.040
Infant 2	3–5 years	3/3/1	3/1/0	12	12	6	0.021–0.041
Juvenile	6–11 years	2/1/1	3/1/1	9	9	7	0.024–0.080
Adult	12^+^ years	4/5/0	4/5/0	18	18	18	0.020–0.080

### 2.2 Age categories

All individuals within the mountain gorilla sample were of known age. Age of the western lowland gorilla individuals was estimated using dental eruption times and assigned to a specific age category following Smith et al. (1994). Five age categories were defined based on skeletal development and the locomotor transitions reported by Doran (1997) (Figure 1). *Neonate*: from birth to 6 months, the period before significant independent locomotion commences, when individuals are carried by the mother. *Infant 1*: from 7 months to 2.9 years, when independent locomotion has commenced but is characterised by a high degree of arboreal and suspensory behaviours. Terrestrial locomotion consists of a mix of palmigrade and knuckle‐walking quadrupedal locomotion and loading is forelimb dominated (Doran, 1997; Ruff et al., 2013). *Infant 2*: from 3 to 5.9 years, when individuals are still dependent on the mother but are shifting to knuckle‐walking as the main form of terrestrial locomotion. Suspensory behaviours decrease but are still much higher than in older individuals. *Juvenile*: 6–11.9 years, when locomotion is hind‐limb dominant and knuckle‐walking becomes the main form of locomotion. During this period, complete fusion of the epiphysis has yet to occur. *Adult*: 12+ years, when terrestrial knuckle‐walking has become the main form of locomotion and the frequency of arboreal behaviours is adult‐like. During this period, fusion of the epiphysis is complete, although a remnant epiphyseal line may still be visible in the internal bone structure.

Since detailed ontogenetic behavioural data are only available for mountain gorillas (Doran, 1997), we assume that similar transitions in locomotor behaviour occur during western lowland gorilla development, but likely with an increased frequency of arboreality, particularly in later developmental stages (i.e. Juvenile and Adult) (Remis, 1995). Infant individuals of both species have been described as highly arboreal (Ruff et al., 2013, 2018). As the locomotor transition from highly arboreal to mainly terrestrial is more pronounced in mountain gorillas than western lowland gorillas, the former is used to define the age categories. Importantly, we also note that age categories above based on changes in locomotor behaviours and epiphyseal fusion are not necessarily consistent with other developmental changes documented in Virunga mountain gorillas specifically, which reach independence (e.g., weaning at ~3.5 years; Eckardt et al., 2016) and age at first birth (~8+ years; Williamson & Gerald‐Steklis, 2001) earlier than Bwindi mountain gorillas and western lowland gorillas (Robbins & Robbins, 2021).

### 2.3 Scan acquisition

Micro‐tomographic scans (microCT) were collected using NIKON XTH225 ST scanners housed at the Cambridge Biotomography Centre, Department of Zoology, University of Cambridge (UK) and at the Shared Materials Instrumentation Facility at Duke University (USA), and using the Diondo D3, and SkyScan1173 scanners housed at the Department of Human Evolution, Max Planck Institute for Evolutionary Anthropology (Germany). Scan parameters were 100–160 kV and 70–190 μA, using a brass or copper filter of 0.25–0.50 mm. Voxel resolution was influenced by individual specimen size, and ranged from 19 to 45 microns. Three specimens (one 8 years of age, two adults) scanned at 80 microns, and six specimens (all adults) at 50–60 microns were included in the sample. The trabecular parameter values of these individuals were compared to the remaining adult sample scanned at 30–40 microns and were not found to be statistically significantly different and thus these specimens were included in all analyses. All scans were reconstructed as either 16‐bit or 8‐bit TIFF image stacks.

### 2.4 Segmentation

All specimens were reoriented into a standardised anatomical position in Avizo 6.3 (FEI Visualisation Sciences Group). In specimens where the epiphysis was attached but not yet fully fused to the distal metaphysis, the two skeletal regions were separated manually in Avizo 6.3 and saved as separate TIFF image stacks. All scans were segmented using the mia‐clustering method (Dunmore et al., 2018).

**FIGURE 2:**
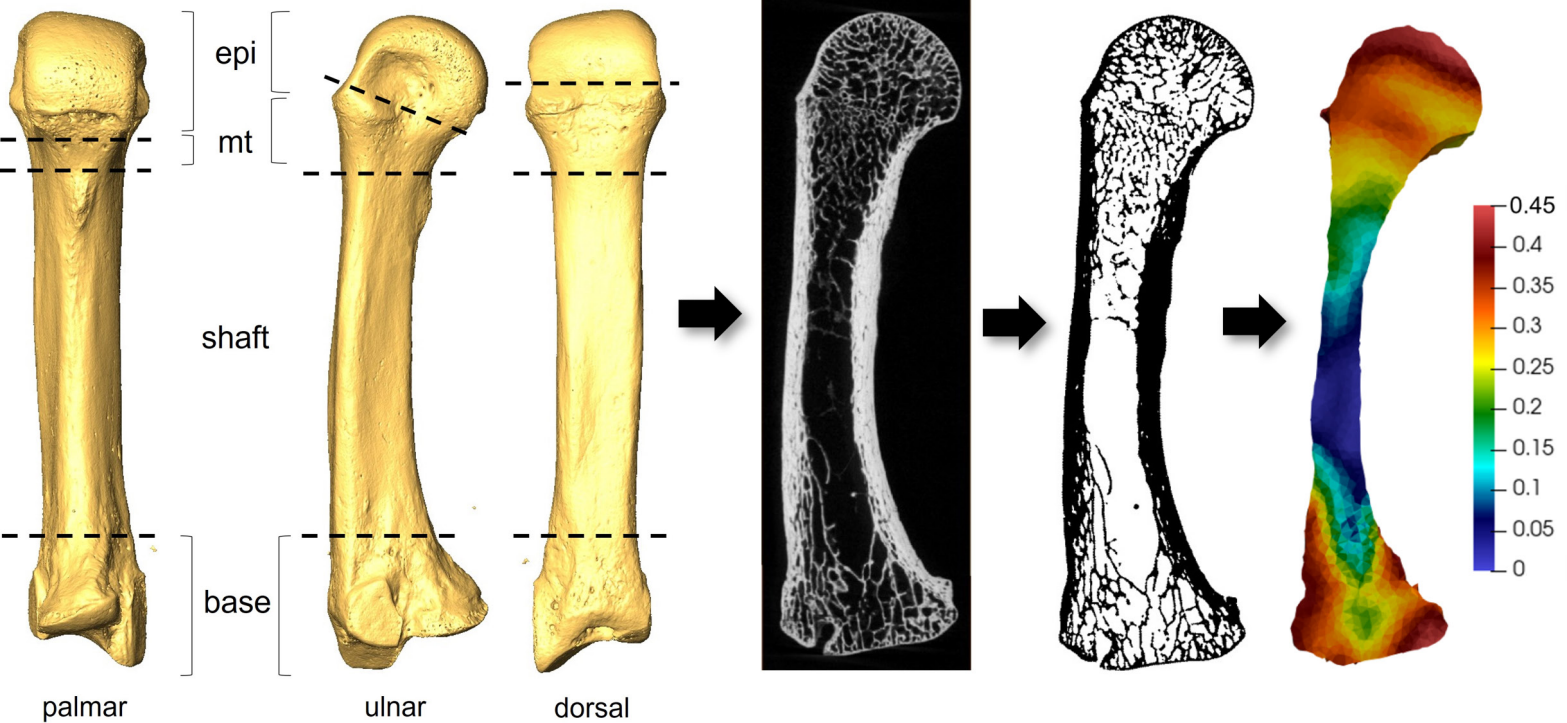
Methodological steps involved in holistic trabecular bone analysis. From left to right: *Gorilla* third metacarpal surface model generated from microCT scan, depicting anatomical boundaries of the base, shaft, metaphysis (mt) and epiphysis (epi); midsagittal cross‐section of raw microCT data showing internal trabecular structure; segmented midsagittal cross‐section; and 3D mesh model with colour map showing BV/TV as per scale

A holistic morphometric analysis was used to analyse the trabecular structure in the metacarpal base, distal metaphysis and epiphysis (Figure 2). The cortical shell, trabecular bone and internal region were segmented using an in‐house script for medtool 4.4 (www.dr‐pahr.at), following Gross et al. (2014) and Tsegai et al. (2013), allowing cortical bone and trabecular bone to be analysed separately. Briefly, after segmenting the inner trabecular structure from the outer cortical shell, a 2.5 mm background grid is superimposed on the isolated trabecular volume (the “inner mask”) and overlapping 5 mm spherical volumes of interest placed at each node are used to quantify trabecular parameters. After generation of a tetrahedral mesh of the inner trabecular region, these trabecular variables are interpolated to each element of the mesh, creating a morphometric map of the trabecular region. Mean trabecular values for BV/TV and DA were calculated using mia‐multi and trabecular thickness, separation and number were calculated using histomorph within medtool 4.4 (www.dr‐pahr.at).

As this method analyses the trabecular bone morphology across the whole bone, the in‐house script was adapted to allow for the separate analysis of the base of the metacarpal, the distal metaphysis and fused epiphysis of the third metacarpal. This script cut the scan into three (unfused epiphysis) or four (fused epiphysis) sections (Figure 2). For individuals where fusion of the epiphysis had commenced and the epiphysis and growth plate were no longer clearly separated by a layer of cartilage, a cutting script was used to divide the bone up into four sections: base, shaft, metaphysis, epiphysis. These areas were identified visually on the scans in Avizo 6.3 (FEI Visualisation Sciences Group) and the slice numbers demarcating these regions were included in the cutting script that would be run through medtool 4.4 (www.dr‐pahr.at; see below and Figure 2). The distal border of the base was defined as the point where the shaft became more concave palmarly in ulnar or radial view, just distal to metacarpal and carpal articulations and roughened non‐articular regions of the radial or ulnar sides (depending on which is most distally extended) of the base. This anatomical boundary ensured that trabeculae within the proximal end of the metacarpal were quantified without including a substantial portion of the medullary cavity (Figure 2). The shaft was identified as the area between the distal border of the base and the proximal border of the metaphysis and was not analysed due to absence of or minimal trabeculae in this region. The proximal border of the metaphysis was defined by the start of dorsal‐palmar widening of the shaft in palmar view, at approximately 70% of the total length of metacarpal measured from the proximal end. This boundary typically coincided with the most distal extent of the entheses for the transverse head of the m. adductor pollicis (van Leeuwen et al., 2018). The distal border of the metaphysis was the growth plate. The epiphysis was defined as the metacarpal head, distal to the growth plate. Where fusion of the epiphysis was complete externally, the proximal border of the epiphysis was defined by the remnant epiphyseal line within the trabecular structure, or the oblique CT slice just distal to the dorsal ridge if the epiphyseal line had been obliterated internally.

### 2.5 Quantification of trabecular variables

Five trabecular bone parameters were quantified from the inner mask generated using the in‐house script for medtool 4.4 (www.dr‐pahr.at): BV/TV, DA, trabecular thickness (Tb.Th), number (Tb.N) and separation (Tb.Sp). This script calculates these parameters as follows: BV/TV and DA were calculated as the mean of values in the 3D tetrahedral mesh; and for DA the mean intercept length method was used and values were bound between 0 (isotropic) and 1 (anisotropic). Tb.Th and Tb.Sp were calculated using the sphere fitting method by Hildebrand and Rüegsegger (1997); and Tb.N was calculated as 1/(Tb.Th + Tb.Sp).

### 2.6 Statistical analysis

Non‐parametric methods were employed to test for significant differences in trabecular variables, as data were not normally distributed across the sample according to the Shapiro–Wilk test. All tests were conducted both with species pooled, to test for overall ontogenetic changes in the gorilla third metacarpal throughout life, as well as with species separated to identify any variation in trabecular morphology due to different activity patterns during ontogeny. Kruskal‐Wallis tests were employed to test for differences in trabecular variables between age groups, followed by post hoc Nemenyi tests (both for species pooled and interspecific comparisons). Three ratios for each trabecular variable between the distal metaphysis, epiphysis and base of the third metacarpal were also calculated to test for significant changes in trabecular morphology in these specific regions during ontogeny using Kruskal‐Wallis tests. A *p*‐value of <0.05 was considered significant. For multivariate analysis two principal component analyses (PCA) were conducted using singular value decomposition of the scaled and centred data matrix. These were conducted for the base and metaphysis together and separately for the epiphysis, as an epiphysis was not present for all individuals. All statistical tests were conducted in R v3.6.3 (R Core Team, 2016) and visualised using the ggplot2 package (Wickham, 2009).

## 3 RESULTS

### 3.1 Ontogenetic changes in *Gorilla* trabecular structure

**FIGURE 3:**
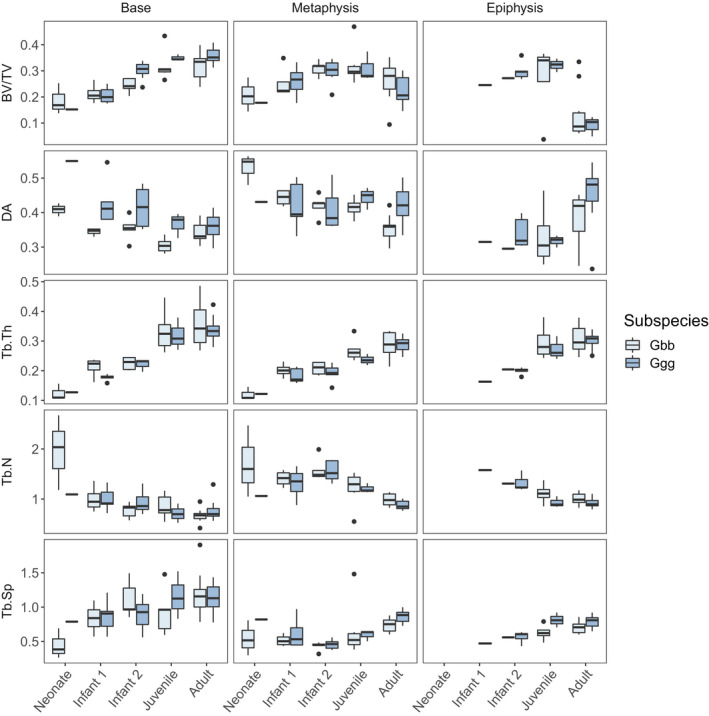
Box‐and‐whisker plots of trabecular parameters (raw values) for each metacarpal region and age category in *Gorilla gorilla gorilla* (Ggg) and *Gorilla beringei beringei* (Gbb). See also Table S1

To investigate potential changes in trabecular structure throughout ontogeny, species‐pooled descriptive statistics for each region of the metacarpal are presented in Table S1 and box‐and‐whisker plots of trabecular parameters by age category are shown in Figure 3. Kruskal–Wallis tests show significant differences between age categories for all trabecular parameters, and general patterns of ontogenetic change in the trabecular structure are described below. Post hoc Nemenyi tests further identify that these significant differences mainly occur between the Neonate‐Juvenile, Neonate‐Adult, Infant 1/2‐Juvenile and Infant 1/2‐Adult age categories in the metacarpal base and metaphysis and the Infant 2‐Adult age category in the epiphysis (Tables 2, 3, 4). Bone volume fraction and Tb.Th are lowest at birth and increase gradually with age in the base, metaphysis and the epiphysis until adulthood when BV/TV decreases slightly at the metaphysis and epiphysis. Tb.Sp remains relatively constant across all regions of the metacarpal until approximately 9 years of age, when a slight increase can be observed in adults. In all three regions, Tb.N is high at birth and subsequently decreases with age.

**TABLE 2:** Significant differences (post hoc Nemenyi results) in trabecular parameters between age categories in the *Gorilla* (species pooled) metacarpal base. Significant results (*p* < 0.05) are shown in bold

Variable	Neonate	Infant 1	Infant 2	Juvenile
BV/TV				
Infant 1	0.993			
Infant 2	0.261	0.235		
Juvenile	**0.015**	**0.005**	0.481	
Adult	**0.002**	**0.000**	0.104	0.990
Tb.Th				
Infant 1	0.874			
Infant 2	0.402	0.860		
Juvenile	**0.001**	**0.002**	**0.029**	
Adult	**0.000**	**0.002**	**0.001**	0.994
Tb.N				
Infant 1	0.736			
Infant 2	0.183	0.753		
Juvenile	0.063	0.371	0.949	
Adult	**0.003**	**0.017**	0.282	0.853
Tb.Sp				
Infant 1	0.690			
Infant 2	0.183	0.810		
Juvenile	0.169	0.762	1.000	
Adult	**0.004**	**0.033**	0.342	0.557
DA				
Infant 1	0.639			
Infant 2	0.640	1.000		
Juvenile	**0.008**	0.101	0.054	
Adult	0.069	0.563	0.415	0.667

**TABLE 3:** Significant differences (post hoc Nemenyi results) in trabecular parameters between age categories in the *Gorilla* (species pooled) metacarpal metaphysis. Significant results (*p* < 0.05) are shown in bold

Variable	Neonate	Infant 1	Infant 2	Juvenile
BV/TV				
Infant 1	0.590			
Infant 2	**0.036**	0.408		
Juvenile	0.052	0.496	1.000	
Adult	0.712	0.993	0.083	0.151
Tb.Th				
Infant 1	0.723			
Infant 2	0.534	0.998		
Juvenile	**0.005**	**0.039**	0.056	
Adult	**0.000**	**0.000**	**0.000**	0.814
Tb.N				
Infant 1	1.0000			
Infant 2	0.9091	0.765		
Juvenile	0.9589	0.918	0.231	
Adult	0.066	**0.004**	**0.000**	0.082
Tb.Sp				
Infant 1	0.690			
Infant 2	0.183	0.810		
Juvenile	0.169	0.762	1.000	
Adult	**0.004**	**0.033**	0.342	0.557
DA				
Infant 1	0.508			
Infant 2	0.173	0.945		
Juvenile	0.416	1.000	0.982	
Adult	**0.009**	0.208	0.598	0.310

**TABLE 4:** Significant differences (post hoc Nemenyi results) in trabecular parameters between age categories in the *Gorilla* (species pooled) metacarpal epiphysis. Significant results (*p* < 0.05) are shown in bold

Variable	Infant 1	Infant 2	Juvenile
BV/TV			
Infant 1			
Infant 2	0.953		
Juvenile	0.933	0.999	
Adult	0.896	**0.031**	**0.012**
Tb.Th			
Infant 1			
Infant 2	0.986		
Juvenile	0.357	0.064	
Adult	0.161	**0.001**	0.828
Tb.N			
Infant 1			
Infant 2	0.977		
Juvenile	0.349	0.082	
Adult	0.171	**0.003**	0.875
Tb.Sp			
Infant 1			
Infant 2	0.964		
Juvenile	0.398	0.158	
Adult	0.240	**0.015**	0.944
DA			
Infant 1			
Infant 2	1.000		
Juvenile	1.000	0.999	
Adult	0.700	0.154	0.168

**FIGURE 4:**
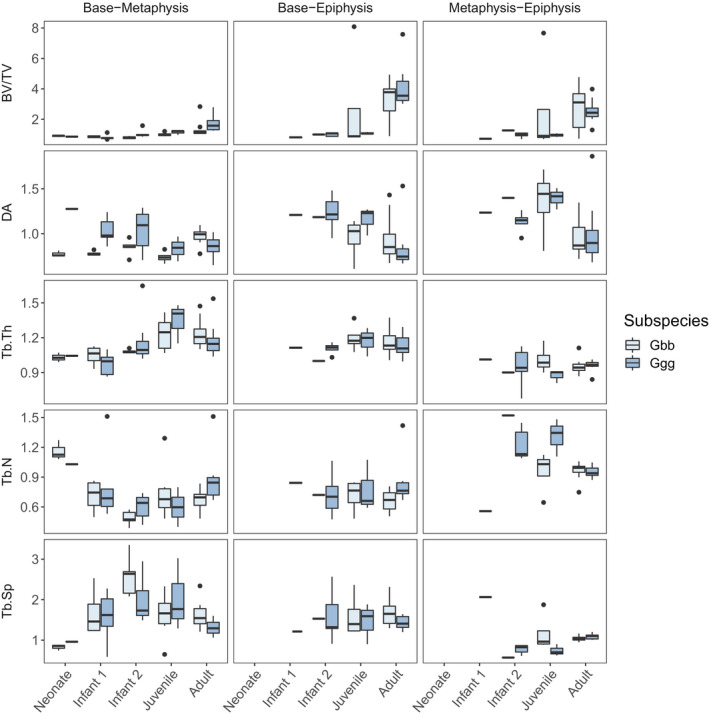
Box‐and‐whisker plots of trabecular parameters for three metacarpal ratios across age categories in *Gorilla gorilla gorilla* (Ggg) and *Gorilla beringei beringei* (Gbb)

The ratio of each trabecular variable between the base, metaphysis and epiphysis of the third metacarpal is shown by age group in Figure 4. Kruskal–Wallis and post hoc Nemenyi tests found significant differences in trabecular ratios only between the Infant 1‐Adult and Juvenile‐Adult age categories. Overall, ratios between the base‐epiphysis and the metaphysis‐epiphysis remain similar throughout ontogeny for Tb.N, Tb.Sp and Tb.Th. BV/TV ratios remain similar in all bone sections until adulthood, when there is higher BV/TV in the base and metaphysis compared to the epiphysis. DA ratios between the base and metaphysis remain relatively constant through early development and then show a slight decrease in anisotropy in adulthood, indicating that the distal metaphysis (i.e. the region that used to be the growth plate) in adulthood is more anisotropic than the base of the gorilla third metacarpal.

**FIGURE 5:**
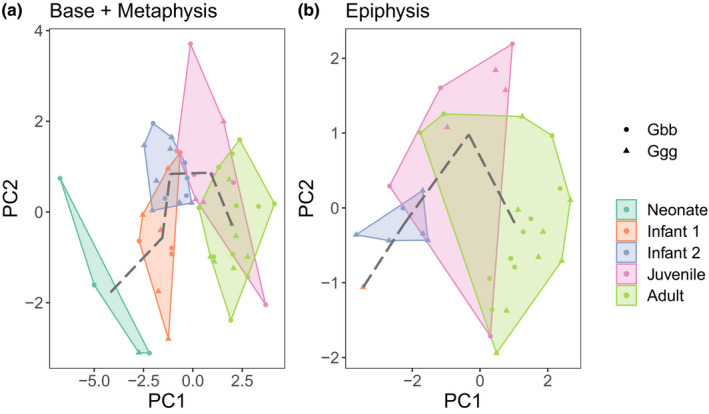
Bivariate plots of first two principal components (PC) for the base and metaphysis (a) and epiphysis (b) analyses. Dotted line connects the mean PC values for each age category

The PCA shows separation between age groups for the base and metaphysis (Figure 5a) and the epiphysis (Figure 5b). Results of both PC analyses are shown in Table 5. For the base and metaphysis, age groups are distributed along PC1, with older individuals (i.e. higher PC1 scores) characterised by higher BV/TV values in the base, and in both the base and metaphysis, lower DA, thicker, more separated and less numerous trabecular struts. For the epiphysis, PC1 also reflects differences between age groups, such that with increasing age there is a reduction in BV/TV and an increase in DA, with trabecular struts also becoming thicker, more separated and less numerous.

**TABLE 5:** Principal component analysis loadings of each trabecular parameter in the third metacarpal base and metaphysis together, and of the epiphysis

	Proportion of variance	Region	BV/TV	DA	Tb.Th	Tb.N	Tb.Sp
Base + Metaphysis							
PC1	0.47	Base	0.27	−0.24	0.43	−0.36	0.33
		Metaphysis	0.00	−0.20	0.43	−0.37	0.29
PC2	0.19	Base	0.36	−0.14	0.13	−0.05	0.01
		Metaphysis	0.66	−0.09	0.13	0.37	−0.48
Epiphysis							
PC1	0.65	Epiphysis	−0.40	0.33	0.47	−0.52	0.49
PC2	0.23	Epiphysis	0.53	−0.68	0.28	−0.29	0.31

### 3.2 Interspecific differences in trabecular ontogeny

Due to the small sample size of the Neonate age category, this category is merged with the Infant 1 age category and potential species differences in the epiphyses are not tested due to the absence of an epiphysis for many individuals in the Infant 1/2 categories (Table 6). The overall trends in ontogenetic changes in all trabecular parameters reported above for the pooled sample remain similar when species are analysed separately (Figure 3). Although DA follows the same pattern of change in both species as observed when species were pooled, there are some significant interspecific differences*. G. g. gorilla* have significantly higher DA in the metacarpal base in the Neonate/Infant 1 and Juvenile categories, and significantly higher DA in the metaphysis in Adults compared with *G. b. beringei* (Table 6; Figure 3). The adult *G. g. gorilla* metaphysis also has significantly lower Tb.N and higher Tb.Sp. relative to *G. b. beringei*, and although *G. g. gorilla* mean BV/TV is lower, this difference is not significant. BV/TV is only significantly different between species in the Infant 2 metacarpal base, with *G. g. gorilla* being significantly higher than *G. b. beringei* (Table 6).

**TABLE 6:** Interspecific differences (post hoc Nemenyi results) in trabecular parameters between age categories in *Gorilla gorilla gorilla* and *Gorilla beringei beringei*. Significant results (*p* ≤ 0.05) are shown in bold. Significant differences were not tested for non‐adult age categories in the epiphysis due to small sample size

Variable/age	Base	Metaphysis	Epiphysis
BV/TV			
Neonate+Infant 1	0.775	0.668	–
Infant 2	**0.019**	0.935	–
Juvenile	0.121	0.606	–
Adult	0.091	0.091	0.722
Tb.Th			
Neonate+Infant 1	1	0.775	–
Infant 2	0.685	0.371	–
Juvenile	1	0.121	–
Adult	0.859	0.859	1
Tb.N			
Neonate+Infant 1	0.317	0.391	–
Infant 2	0.168	0.935	–
Juvenile	0.606	0.302	–
Adult	0.374	**0.041**	0.155
Tb.Sp			
Neonate+Infant 1	0.253	0.317	–
Infant 2	0.223	0.569	–
Juvenile	0.302	0.302	–
Adult	0.789	**0.041**	0.062
DA			
Neonate+Infant 1	**0.046**	0.199	–
Infant 2	0.088	0.569	–
Juvenile	**0.039**	0.197	–
Adult	0.286	**0.009**	0.062

### 3.3 Qualitative trabecular patterns during ontogeny

**FIGURE 6:**
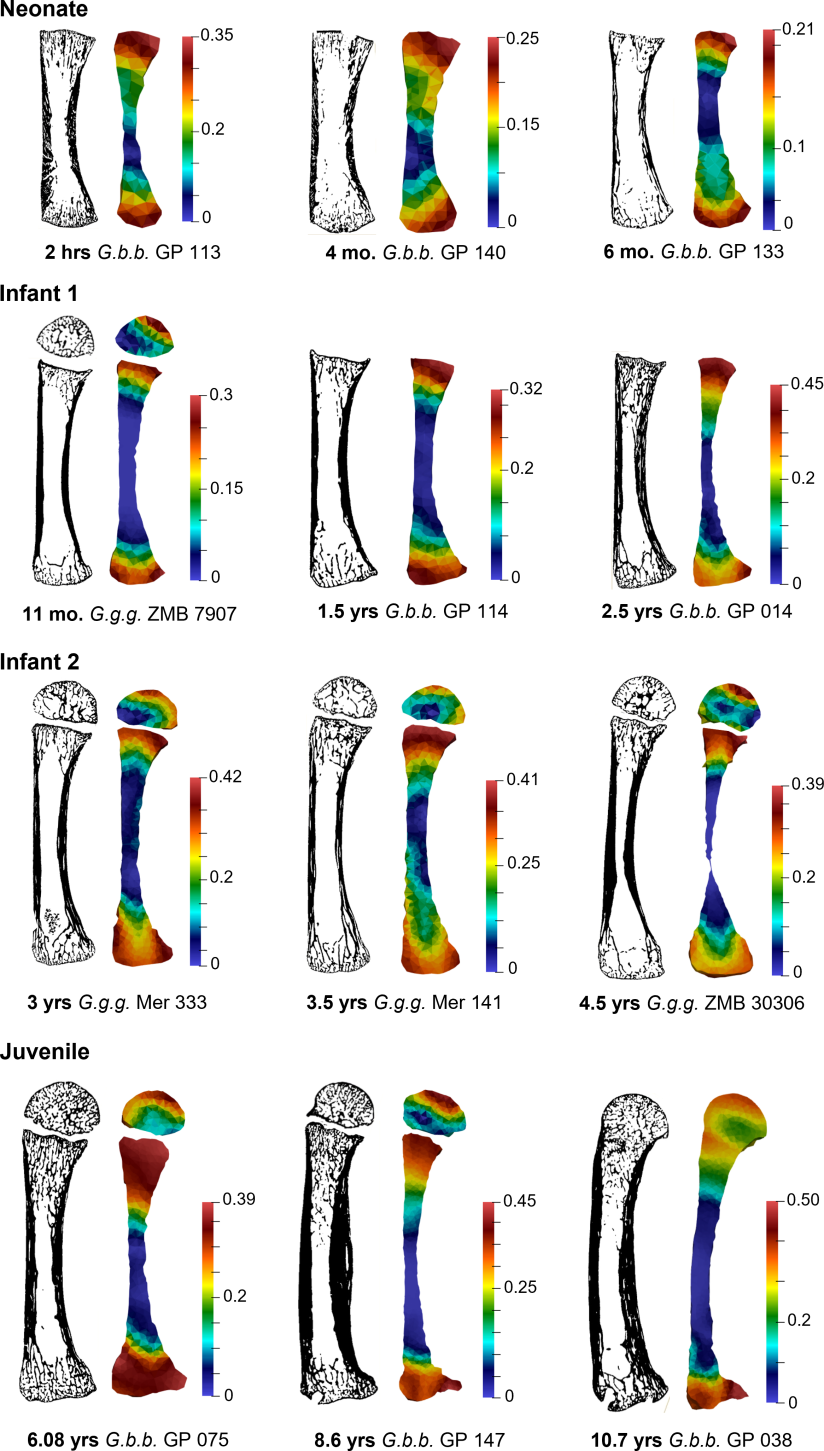
Ontogenetic changes in BV/TV distribution from neonate to juvenile age categories in the *Gorilla* (species pooled) third metacarpal. Each specimen is shown in the midsagittal cross‐section, including segmented trabecular and cortical bone (left) and the BV/TV distribution colour map scaled to the range of each specimen (right). Note epiphyses are often not ossified or preserved in the youngest age categories. Specimen size not to scale. See Figure S1 for colour maps of the same specimens scaled to the same 0–0.45 BV/TV scale and Figure S2 for additional juvenile specimens

**FIGURE 7:**
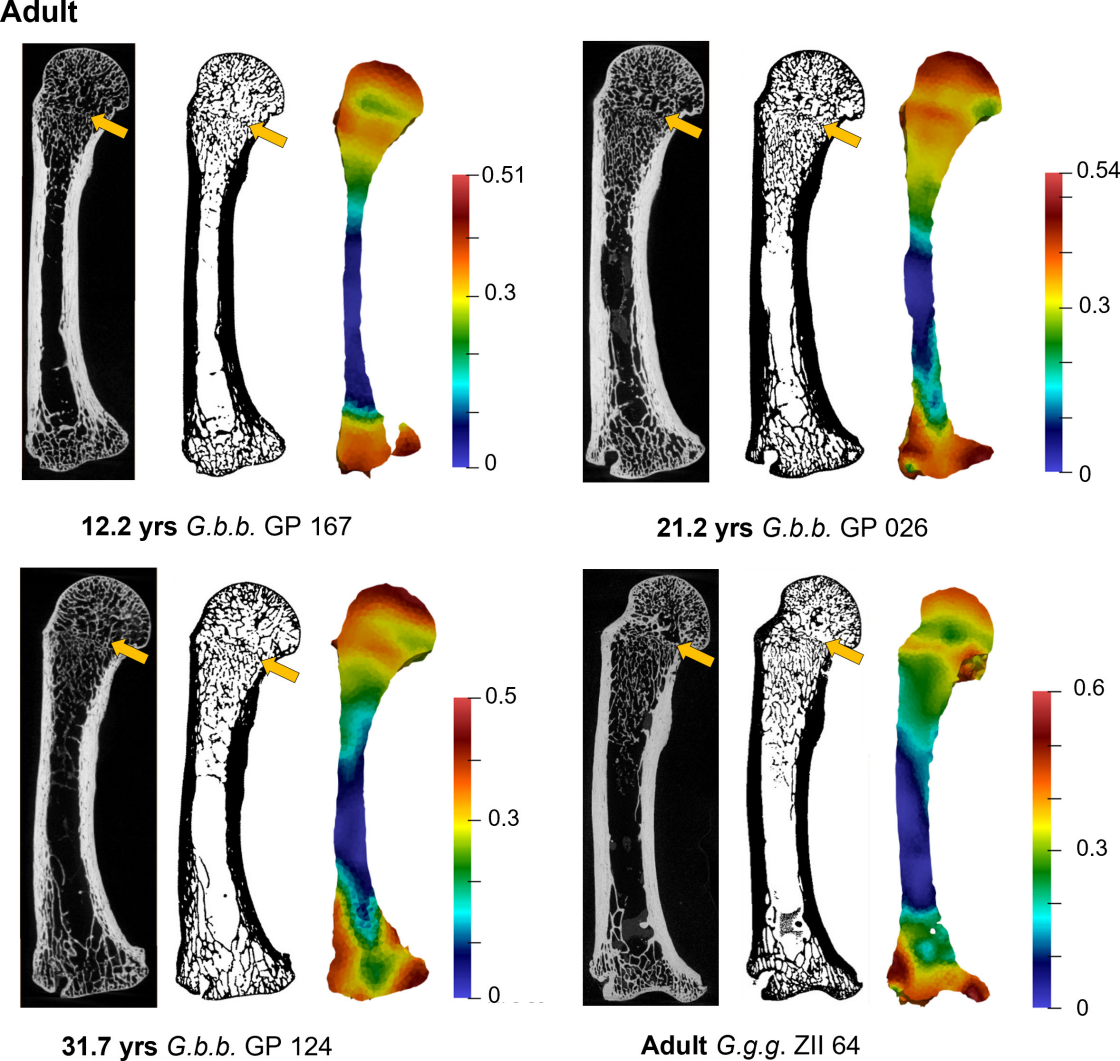
Adult BV/TV distribution in the *Gorilla* (species pooled) third metacarpal. Each specimen is shown in the midsagittal cross‐section, including raw microCT data (left), segmented trabecular and cortical bone, and BV/TV distribution colour map scaled to the range of each specimen (right). Arrow highlights occurrence of remnant epiphyseal line within the trabecular structure. *Gorilla gorilla gorilla* specimen PCM ZII 64 is, based on dental wear, of mid‐range age within our sample. Specimen size not to scale. See Figure S3 for additional adult specimens

Cross‐sections of segmented microCT data and colour maps of the distribution of BV/TV in a subset of the *Gorilla* sample (species pooled) are depicted for non‐adult age categories (Figure 6 and Figures S1 and S2) and adults (Figure 7 and Figure S3). BV/TV is variable at the metacarpal base and metaphysis in the Neonate category (the epiphysis is not preserved for this age group), with relatively high BV/TV just after birth (though slightly lower than that found in adults) but decreasing by 6 months of age. The Infant 1 and 2 categories (≤5 years old) are characterised by high BV/TV underneath the growth plate and at the palmar and dorsal regions of the base. Within the epiphysis, overall BV/TV is low in the Infant 1 and 2 categories when compared to older individuals (Figure S1), although when scaled to the individual BV/TV range, a distopalmar concentration of high BV/TV is observed. During the Juvenile stage (6–11 years), BV/TV remains highest in the metacarpal base and distal metaphysis but at the later stages (i.e., 10–11 years old) the high BV/TV underneath the growth plate changes from being uniformly distributed to being separated into a dorsal and palmar concentrations (Figure 6 and Figure S2). BV/TV in the epiphysis increases at the Juvenile stage, but still does not fully resemble the Adult distribution pattern. Bone volume fraction is highest distally but generally remains high throughout most of the epiphysis. As fusion commences around 10–11 years, the palmar and distal BV/TV concentrations underneath the growth plate and within the epiphysis remain high until full fusion of the cortex has occurred and the epiphyseal line has been mostly resorbed. At this late stage of juvenile development, there is a shift to an adult‐like distodorsal concentration of high BV/TV within the epiphyses and low BV/TV in the proximal portion of this region.

Among our Adult sample, high BV/TV in the base is concentrated in the palmar and dorsal regions (Figure 7 and Figure S3). Within the distal metaphysis, BV/TV is generally higher palmarly, while within the metacarpal head, BV/TV is concentrated distodorsally, creating a low BV/TV region within the proximopalmar region of the head. Interestingly, most adult specimens (60% of *n* = 10 females and 100% of *n* = 8 males) in our sample retain a remnant of an epiphyseal line. This is typically represented as a notable difference in the trabecular structure at the growth plate region, with the retention of thinner, more numerous trabeculae at the distal boundary of the metaphysis contrasted with thicker, less numerous trabeculae at the proximal broadening of the epiphysis (Figure 7 and Figure S3). This remnant epiphyseal structure is maintained in *G. b. beringei* individuals as old as 39 years, and possibly older *G. g. gorilla* (Figure S3).

## 4 DISCUSSION

This study aimed to provide greater insight into trabecular changes in the gorilla third metacarpal throughout ontogeny and its potential correlation to shifts in locomotor patterns with age. Understanding how immature activity patterns influence adult trabecular bone provides further insight into the functional interpretations we can draw from adult great ape trabecular morphology and more robust reconstructions of behaviour in fossil hominins.

### 4.1 *Gorilla* ontogenetic changes

We predicted that ontogenetic changes in trabecular structure would follow the patterns previously identified in chimpanzees (Ragni, 2020; Tsegai et al., 2018), with BV/TV gradually increasing throughout ontogeny and with DA remaining constant within the base but becoming more anisotropic within the metacarpal head. These predictions were generally supported by our results. We found that gorilla trabecular bone structure in the Neonate category was categorised by thin, closely spaced trabeculae, with low BV/TV compared to older age groups. As age progressed, BV/TV and Tb.Th increased in all bone sections, while Tb.Sp remained relatively constant throughout ontogeny. Trabecular number was high in Neonates and decreased throughout ontogeny in all regions. Significant differences between age categories for most trabecular variables were mainly between the Neonate‐Infant 1 categories relative to the Juvenile‐Adult categories. As the Juvenile age category is marked by the full adoption of knuckle‐walking and more adult‐like activity patterns, it may be that these trabecular changes reflect this shift from more variable locomotor behaviours to consistent loading during frequent quadrupedalism (Figure 1).

When comparing our results to other ontogenetic studies of non‐human ape (Ragni, 2020; Tsegai et al., 2018; Zeininger, 2013) and human (Gosman & Ketcham, 2009; Milovanovic et al., 2017; Perchalski et al., 2018; Raichlen et al., 2015; Ryan & Krovitz, 2006) trabecular bone, we found both similarities and differences in the ontogenetic pattern of trabecular bone in the gorilla third metacarpal. In humans, BV/TV and Tb.N (or connectivity density) in the proximal femur (Milovanovic et al., 2017; Ryan & Krovitz, 2006), proximal tibia (Gosman & Ketcham, 2009) and calcaneus (Saers et al., 2020) were higher in perinatal humans than at any other stage of life (Nuzzo et al., 2003). Between six to 12 months, BV/TV and Tb.N dramatically reduced but then increased when independent bipedal locomotion began around 1.5–2 years of age, although only in the calcaneus did they reach the same levels as seen during the perinatal phase (Gosman & Ketcham, 2009; Ryan & Krovitz, 2006; Saers et al., 2020). This decrease in BV/TV and Tb.N in early childhood may be related to the advent of bipedal locomotion in humans, however, this decrease was also observed in the modern human and Neandertal humerus (Chevalier et al., 2021; Ryan et al., 2017), suggesting a combination of genetic and mechanical factors are influencing systemic patterns of trabecular ontogeny.

The pattern of trabecular ontogeny among great apes differs in some aspects from that of humans. In the great ape calcaneus (Zeininger, 2013), the chimpanzee humerus, femur, and tibia (Tsegai et al., 2018), and the chimpanzee and gorilla third metacarpal base (Ragni, 2020), BV/TV and Tb.Th were lowest at birth and increased throughout ontogeny, without a decrease occurring at 6 months of age or any other juvenile period. Our results on the gorilla metacarpal showed a similar pattern to that of the African ape forelimb (Ragni, 2020; Tsegai et al., 2018), with low BV/TV and Tb.Th at birth and both increasing throughout ontogeny. Both humans and great apes share a pattern of increasing Tb.Th throughout ontogeny (Gosman & Ketcham, 2009; Ryan et al., 2017; Ryan & Krovitz, 2006) that may be correlated with body size changes in the proximal tibia (Gosman & Ketcham, 2009), but not in the calcaneus (Saers et al., 2020). This increase in thickness suggests that bone deposition is occurring to reinforce trabecular struts and, at the same time, an increase in the size of the growing region (metaphysis/base/epiphysis) leads to these trabeculae being less densely spaced (i.e. increase in Tb.Sp and reduction in Tb.N).

In contrast to Tb.Th, our results and those of Tsegai et al. (2018) suggest that the pattern of BV/TV ontogeny in great apes differs from that of humans in lacking a decrease in BV/TV during the first year of life. This difference suggests variation in the rate of increased thickness and the rate of increased separation between trabecular struts. However, although the youngest individual in our sample (2 h old) does have a low BV/TV when compared to older age categories, it has higher BV/TV than older individuals in the Infant 1 age category (those aged 2 weeks and older).

There may be several reasons for this documented ontogenetic variation in trabecular morphology across species. The high density of trabecular bone in humans may be due to genetic regulation (Acquaah et al., 2015), which may also be the case for other primate species. Although there is movement of the foetus in utero, it is possible that it is not until after birth that mechanical loading will have an influence on trabecular bone structure. Furthermore, it may be more effective to remodel a dense trabecular structure by removing bone in response to mechanical loading than it is to create new struts in a sparsely populated trabecular structure (Tanck et al., 2001; Tsegai et al., 2018). Variation in life history likely also plays an important role given that precocial species need to load their limbs immediately after birth. For example, the trend of high BV/TV at birth with a decrease as locomotion commences that was found in humans was also found in the limb bones of altricial domestic dogs (Wolschrijn & Weijs, 2004), while precocial horses, which are able to walk and run within hours of birth, showed high BV/TV at birth but no subsequent decrease (Gorissen et al., 2018). Thus, the consistently high BV/TV found in precocial species may be a result of a shift in timing in which the BV/TV decrease actually happens in utero and the trabecular structure is subsequently predetermined to anticipate the mechanical loading that will occur after birth (Gorissen et al., 2016, 2018; Tsegai et al., 2018). This latter explanation, however, is unlikely to explain the difference in BV/TV observed in the gorilla metacarpal between individuals that were 2 h and 2 weeks of age, as during this time the infants are wholly dependent on and carried by their mothers (Doran, 1997). The high BV/TV in the 2 h‐old individual may be pathological (given its early death) or simply individual variation. More individuals at similarly young age are necessary to ascertain if this is an aberration in the data or a developmental pattern in *Gorilla*.

Our prediction that DA would remain relatively constant throughout ontogeny within the metacarpal base but would increase within the epiphysis from 5 years of age as habitual loading during knuckle‐walking increased was partially supported. We found an intermediate DA (i.e. a mix of isotropic and anisotropic trabeculae) that remained relatively constant throughout ontogeny in the base, distal metaphysis and epiphysis (Table S1; Figure 3). Although there was a notable increase in DA in the epiphysis between the Juvenile and Adult age categories (Table S1; Figure 3), this difference was not significant (Table 4). This result is consistent with Ragni’s (2020) findings in the base of the African ape third metacarpal, and was expected for the region of the metaphysis/growth plate before fusion occurred as the deposition of primary trabeculae during bone growth would be uniform until remodelled into secondary trabeculae under the influence of mechanical loading (Scheuer et al., 2000). In contrast to our results, a reduction in DA reflecting more variable joint loads was observed in the infant chimpanzee humerus and tibia before increasing again at the juvenile stage (>5 years of age) (Tsegai et al., 2018). This pattern was also found in the humerus and lower limb of modern humans, where DA decreased when independent locomotion commences (6 months to 1 year) and increased as bipedality became habitual (Gosman & Ketcham, 2009; Perchalski et al., 2017; Raichlen et al., 2015; Ryan & Krovitz, 2006). The relatively constant DA across all regions of the gorilla metacarpal throughout ontogeny that we found may reflect differences in locomotion and variation in hand postures (Neufuss et al., 2017; Thompson et al., 2018) that result in an ‘intermediate DA’ pattern. Alternatively, since our method analyses the whole structure and not a volume of interest located near an articulation, it may be that there are differences in DA subchondrally but that these are being averaged out by opposite DA values found elsewhere in the bone.

### 4.2 Trabecular bone distribution

Previous research revealed a high concentration of BV/TV in the distodorsal region of the metacarpal head that has been linked to habitual loading of an extended metacarpophalangeal joint during knuckle‐walking in adult gorillas and, to a lesser extent, more arboreal chimpanzees and bonobos (Dunmore et al., 2019; Matarazzo, 2015: Tsegai et al., 2013). Therefore, we predicted that if BV/TV distribution similarly reflected changes in presumed locomotor hand postures during ontogeny, then (1) these functional signals would be stronger in the epiphysis compared to that of the base and (2) that BV/TV would be highest in the palmar region of the epiphysis in early ontogeny, reflecting more flexed‐finger grasping and would shift to higher BV/TV dorsally as knuckle‐walking became the dominant form of locomotion. This prediction was partially supported.

During the first 6 months of life, BV/TV was fairly homogenously distributed compared to older individuals but showed higher BV/TV concentrations underneath the growth plate and in the dorsal and palmar aspects of the base. No data were available for the epiphysis for this age category. As independent locomotion is minimal at this age, it is more likely that the higher concentrations of BV/TV, especially at the growth plate, are due to general changes in morphology due to growth (Rolian, 2016; Scheuer et al., 2000) rather than biomechanical changes related to behaviour.

From 11 months to 6 years of age (Infant 1 to Infant 2), BV/TV remained high at the growth plate and metacarpal base and was generally homogenously distributed dorsopalmarly within these regions (Figure 6 and Figure S1). BV/TV was low in the epiphysis relative to the metaphysis and base, but with the highest BV/TV concentrations distopalmarly. As palmigrade and suspensory behaviours and use of arboreal substrates are still frequent during this period (Doran, 1997), higher distopalmar concentrations in the epiphysis may reflect greater use of flexed metacarpophalangeal joint postures.

We found notable changes in the distribution of BV/TV throughout the Juvenile stage (6–11.9 years) (Figure 6 and Figure S2). The metacarpal base showed an increase in BV/TV due to a thickening of the trabeculae (Figure 3), which may also reflect increased body size, but BV/TV was concentrated in the palmar and dorsal regions of the base. Within the metaphysis, BV/TV distribution shifted from being high throughout the region of the growth plate (6–8 years) to low at the centre of the growth plate (10–11 years). This pattern most likely reflects the beginning of epiphyseal fusion; as trabeculae at the centre of the epiphyseal line are resorbed, more load needs to be distributed through the trabeculae in the dorsal and palmar sections. Within the epiphysis, BV/TV was high distally (and low palmarly), but throughout this Juvenile stage, BV/TV also increased in the dorsal region, particularly in older individuals (10–11 years). It is not until after full fusion of the epiphysis (12+ years) that the adult‐like BV/TV distribution pattern emerges (in both *G. g. gorilla* and *G. b. beringei*), in which BV/TV concentrations are highest distodorsally in the metacarpal head and, although less consistently across individuals, palmarly in the base.

The fact that an adult‐like BV/TV distribution in the third metacarpal does not appear until 12+ years of age suggests there is an adaptive lag in the bone’s response to adult‐like locomotor patterns. Although locomotor behaviours and substrate use are more diverse between 2–6 years of age compared with adults, knuckle‐walking is the dominant form of locomotion throughout this developmental period (67%–94% of total locomotor time in *G. g. beringei*; Doran, 1997). This knuckle‐walking dominance was partly reflected in higher BV/TV in the dorsal epiphysis of some, but not all, Juvenile individuals but is not consistently present until approximately 10 years *after* knuckle‐walking becomes the dominant form of gorilla locomotion. Such an adaptive lag has been observed in the trabecular development of suids, where peak bone density was reached many weeks before DA reached its adult‐like configuration (Tanck et al., 2001). Evidence of adult trabecular morphology reflecting juvenile behaviour has also been found in the human calcaneus, in which men who had exercised regularly during childhood, but had since stopped this behaviour in adulthood, showed a higher degree of bone mineral density than the adult calcaneus of men who had never exercised during childhood (Pettersson et al., 2010). This suggests that juvenile behaviours influence adult trabecular morphology in great apes as well as in humans (e.g. Anderson et al., 2020).

This disconnect between changes in locomotor behaviour and BV/TV distribution may also be explained, at least in part, by bone growth processes, such that BV/TV remains high along the growth plate as ossification is taking place, which may influence the manner in which loads are distributed through the epiphysis and metaphysis and/or how quickly trabeculae may (re‐)model in other regions of the metacarpal. It is interesting that most adult individuals in our sample retained a remnant of the epiphyseal line within the trabecular structure, some as old as 39 years of age (Figure 7 and Figure S3). These individuals retained a higher concentration of BV/TV where the growth plate used to be and had an overall higher BV/TV in their metacarpal heads than in individuals where the epiphyseal plate had been completely remodelled. Perhaps higher BV/TV within the epiphysis limits the stress incurred by this central region of the distal metacarpal such that the stress is not sufficient to cause modelling of this region.

### 4.3 Interspecific comparisons

Our prediction that there would be significant differences between species in the trabecular structure due to changes in activity patterns after 5 years of age was not supported. Although we did find significant differences between *G. g. gorilla* and *G. b. beringei* in DA in the metacarpal base early in ontogeny and in the metaphysis within Adults that may be indicative of slightly different loading patterns, there were no significant differences in the other bone regions, particularly the epiphysis, as we predicted. While adult western lowland gorillas have been reported to be more arboreal than mountain gorillas (Doran, 1997; Remis, 1995, 1998), their overall locomotor patterns remain similar. Both practice terrestrial knuckle‐walking as their main form of locomotion, interspersed with vertical climbing and other arboreal behaviours (Crompton et al., 2010; Hunt, 2004). These species differences in locomotion have been shown to be reflected in the overall morphology of the talus (Dunn et al., 2014; Knigge et al., 2015) and in the cortical geometry of the fore‐ and hindlimb (Ruff et al., 2013, 2018). The lack of difference observed between species in this study may indicate that trabecular bone responds to different types and directions of mechanical loading (together with metacarpal cortical bone) but is not sensitive enough to reflect slight differences in frequency of these behaviours. Alternatively, differences in locomotor loading may be accommodated primarily within changes to cortical bone structure throughout ontogeny instead of trabeculae. Ruff et al. (2013, 2018) showed that the cross‐sectional geometry of mountain and western lowland gorilla fore‐ and hind limb long bones reflected changes in their locomotion patterns during ontogeny. The forelimb/hindlimb diaphyseal strength ratios were similar in mountain and lowland gorilla individuals under the age of 2 years when both species show higher frequencies of arboreal behaviour but then diverged after this age as mountain gorillas engaged in more terrestrial locomotion (Ruff et al., 2013, 2018). However, a similar study has not yet been done for gorilla metapodials.

### 4.4 Implications for interpreting behaviour in the past

Our study provides new data on trabecular bone response to mechanical loading in juvenile gorillas, which may be used as a comparative framework in the behavioural reconstruction of (juvenile) fossil hominins via trabecular bone parameters. The functional signal within the metacarpal trabecular structure reflecting the transition from a higher frequency of arboreal behaviours to primarily terrestrial locomotion in gorillas observed in this study, albeit not necessarily coincident with the ontogenetic timing of these locomotor changes, indicates that similar locomotor transitions may also be reflected in fossil hominin trabecular architecture. This ability may provide novel insight into the functional significance of external morphology and whether climbing or arboreal behaviours were a substantial component of the locomotor repertoires of particular fossil hominin species (Desilva et al., 2018; Green & Alemseged, 2012; Ward, 2002). Furthermore, the adaptive lag observed between adult‐like trabecular patterns and adult locomotor behaviours we observed in the gorilla metacarpal indicates that we should exercise caution when interpreting behaviour from trabecular bone in young adult fossil hominin specimens, as we may be observing signals of juvenile behaviour if such a lag exists in these species as well.

There are some limitations to our study that should be acknowledged. We assume that more frequent behaviours, such as knuckle‐walking and climbing, would be better reflected by the trabecular morphology of the third metacarpal than infrequent behaviours (Rubin et al., 2002). As there were no behavioural data available for the specimens in our study sample, we assumed that the frequency of locomotor behaviours changed throughout ontogeny in the same manner as described by Doran (1997) and that locomotor ontogeny in western lowland gorillas was similar, apart from a higher frequency of arborealism, to mountain gorillas. Detailed investigation of the locomotor ontogeny in multiple species and populations of gorillas is still needed to understand the potential variation and how it relates to species or ecological factors. It must also be remembered that systemic and environmental factors influence trabecular bone structure (Kivell, 2016; Lieberman, 1996; Tsegai et al., 2018). Lastly, this study was limited by small sample sizes for each age category and thus sexes had to be pooled for analyses. As gorillas are a highly sexual dimorphic species (Shea, 1986; Taylor, 1997), there may be differences in trabecular morphology due to differences in body size or behaviour that could be investigated with larger sample sizes.

## 5 CONCLUSION

This study has shown that ontogenetic changes in trabecular architecture, including the distribution of BV/TV, in the gorilla third metacarpal reflect shifts in locomotion throughout development, but that there is a potential adaptive lag between adult locomotion and the appearance of adult BV/TV distribution patterns. However, third metacarpal trabecular structure did not reflect more subtle (presumed) interspecific differences in locomotion between *G. g. gorilla* and *G. b. beringei*. While changes in trabecular structure in the gorilla third metacarpal are similar to those found in the chimpanzee upper limb (Tsegai et al., 2018) and third metacarpal base (Ragni, 2020), the presence of high BV/TV at birth followed by an immediate reduction in BV/TV may indicate that not all skeletal elements in great apes follow the same pattern of response to loading throughout ontogeny. More data should be collected on peri‐natal trabecular bone morphology to identify to what degree early trabecular bone is genetically predetermined or influenced by biomechanical loads.

## AUTHOR CONTRIBUTIONS

This project was conceived by Kim Deckers, Tracy L. Kivell and Matthew M. Skinner. All authors contributed in part to data collection, including Angel Zeininger who scanned all the *G. beringei beringei* specimens. Kim Deckers and Zewdi J. Tsegai performed data analysis and Kim Deckers, Zewdi J. Tsegai, Matthew M. Skinner and Tracy L. Kivell contributed to interpretation of results. Kim Deckers and Tracy L. Kivell drafted the manuscript and all authors contributed critical input and feedback on multiple versions of the manuscript.

## Supporting information


**Appendix** S1

## Data Availability

The trabecular data that support the findings of this study are available from the corresponding author upon request. Availability of microCT data is determined upon request to curators at the host museum.
