# Supplementary material for: Ontogenetic changes to metacarpal trabecular bone structure in mountain and western lowland gorillas
Source: J Anat. 2022 Feb 4;241(1):82–100. doi: 10.1111/joa.13630 (PMC9178373; doi:10.1111/joa.13630)
Supplement: Supplementary file 1 — Appendix S1 [file JOA-241-82-s001.zip › JOA_13630_Table.docx]

**Supplementary Information**

**Table S1.** Mean and standard deviation (in parentheses) of trabecular variables for each age category in different regions of the (A) *Gorilla* (species pooled) (B) *G. g. gorilla* and (C) *G. b. beringei* third metacarpal.

**(A)**

| ***Gorilla*** | **BV/TV (%)** | **Tb.Th (mm)** | **Tb.Sp (mm)** | **Tb.N (mm^-1^)** | **DA** |
| --- | --- | --- | --- | --- | --- |
| **Base** | | | | | |
| Neonate | 17.78 (5.15) | 0.13 (0.02) | 0.53 (0.25) | 1.75 (0.75) | 0.44 (0.07) |
| Infant 1 | 20.97 (3.24) | 0.19 (0.03) | 0.86 (0.22) | 0.99 (0.23) | 0.39 (0.07) |
| Infant 2 | 27.76 (4.30) | 0.22 (0.02) | 0.98 (0.26) | 0.87 (0.20) | 0.39 (0.06) |
| Juvenile | 32.90 (4.93) | 0.33 (0.06) | 1.00 (0.33) | 0.80 (0.23) | 0.33 (0.04) |
| Adult | 33.65 (4.82) | 0.35 (0.07) | 1.17 (0.27) | 0.72 (0.19) | 0.35 (0.03) |
| **Metaphysis** | | | | | |
| Neonate | 19.97 (5.53) | 0.12 (0.02) | 0.61 (0.25) | 1.55 (0.67) | 0.51 (0.06) |
| Infant 1 | 25.69 (5.75) | 0.19 (0.03) | 0.57 (0.17) | 1.35 (0.24) | 0.43 (0.05) |
| Infant 2 | 30.22 (3.91) | 0.19 (0.03) | 0.45 (0.07) | 1.57 (0.21) | 0.41 (0.47) |
| Juvenile | 31.75 (6.62) | 0.26 (0.04) | 0.64 (0.33) | 1.21 (0.28) | 0.42 (0.30) |
| Adult | 24.41 (6.77) | 0.29 (0.04) | 0.80 (0.12) | 0.94 (0.12) | 0.38 (0.06) |
| **Epiphysis** | | | | | |
| Neonate | N/A | N/A | N/A | N/A | N/A |
| Infant 2 | 24.55 | 0.16 | 0.47 | 1.58 | 0.32 |
| Infant 2 | 29.56 (3.36) | 0.20 (0.01) | 0.56 (0.07) | 1.32 (0.14) | 0.33 (0.04) |
| Juvenile | 29.28 (1.15) | 0.29 (0.05) | 0.71 (0.15) | 1.03 (0.19) | 0.33 (0.07) |
| Adult | 11.48 (7.50) | 0.30 (0.04) | 0.75 (0.10) | 0.97 (0.12) | 0.41 (0.09) |

**(B)**

| ***G. g. gorilla*** | **BV/TV (%)** | **Tb.Th (mm)** | **Tb.Sp (mm)** | **Tb.N (mm-1)** | **DA** |
| --- | --- | --- | --- | --- | --- |
| **Base** | | | | | |
| Neonate | 15.22(N/A) | 0.13 (N/A) | 0.79 (N/A) | 1.09 (N/A) | 0.55 (N/A) |
| Infant 1 | 20.69 (3.17) | 0.18 (0.01) | 0.87 (0.24) | 1.00 (0.27) | 0.43 (0.07) |
| Infant 2 | 30.16 (3.52) | 0.22 (0.02) | 0.89 (0.22) | 0.93 (0.22) | 0.42 (0.06) |
| Juvenile | 35.01(1.13) | 0.32 (0.06) | 1.16 (0.35) | 0.71 (0.19) | 0.37 (0.04) |
| Adult | 35.88 (3.27) | 0.34 (0.05) | 1.13 (0.21) | 0.78 (0.24) | 0.36 (0.04) |
| **Metaphysis** | | | | | |
| Neonate | 17.76 (N/A) | 0.12 (N/A) | 0.82 (N/A) | 1.06 (N/A) | 0.43 (N/A) |
| Infant 1 | 25.97 (5.98) | 0.18 (0.03) | 0.62 (0.22) | 1.31 (0.30) | 0.42 (0.07) |
| Infant 2 | 29.77 (4.65) | 0.19 (0.03) | 0.46 (0.07) | 1.56 (0.21) | 0.41 (0.06) |
| Juvenile | 30.93 (5.61) | 0.24 (0.02) | 0.60 (0.08) | 1.21 (0.10) | 0.44(0.03) |
| Adult | 22.28 (5.52) | 0.29 (0.03) | 0.86 (0.10) | 0.88 (0.09) | 0.42 (0.06) |
| **Epiphysis** | | | | | |
| Neonate | N/A | N/A | N/A | N/A | N/A |
| Infant 2 | 24.55 (N/A) | 0.16 (N/A) | 0.47 (N/A) | 1.58 (N/A) | 0.31 (N/A) |
| Infant 2 | 30.04 (3.52) | 0.20 (0.01) | 0.57 (0.08) | 1.32 (0.16) | 0.34 (0.04) |
| Juvenile | 32.21 (2.58) | 0.27 (0.04) | 0.81 (0.11) | 0.93 (0.11) | 0.32 (0.02) |
| Adult | 9.34 (2.68) | 0.30 (0.03) | 0.79 (0.09) | 0.92 (0.10) | 0.45 (0.10) |

**(C)**

| ***G. b. beringei*** | **BV/TV (%)** | **Tb.Th (mm)** | **Tb.Sp (mm)** | **Tb.N (mm-1)** | **DA** |
| --- | --- | --- | --- | --- | --- |
| **Base** | | | | | |
| Neonate | 18.64 (5.95) | 0.12 (0.03) | 0.45 (0.22) | 1.96 (0.75) | 0.41 (0.02) |
| Infant 1 | 21.31 (3.77) | 0.21 (0.03) | 0.84 (0.22) | 1.00 (0.27) | 0.34 (0.01) |
| Infant 2 | 24.20 (2.84) | 0.23 (0.02) | 1.11 (0.27) | 0.77 (0.15) | 0.35 (0.03) |
| Juvenile | 31.84 (5.87) | 0.33 (0.07) | 0.93 (0.32) | 0.85 (0.24) | 0.30 (0.02) |
| Adult | 31.87 (5.25) | 0.36(0.08) | 1.20 (0.31) | 0.67 (0.14) | 0.34 (0.03) |
| **Metaphysis** | | | | | |
| Neonate | 20.71 (6.53) | 0.12 (0.02) | 0.54 (0.26) | 1.71 (0.72) | 0.53 (0.04) |
| Infant 1 | 25.36 (6.36) | 0.20 (0.02) | 0.52 (0.09) | 1.41 (0.16) | 0.44 (0.02) |
| Infant 2 | 30.85 (2.93) | 0.21(0.02) | 0.43 (0.07) | 1.59 (0.23) | 0.42 (0.03) |
| Juvenile | 32.15 (7.55) | 0.27 (0.04) | 0.66 (0.41) | 1.21 (0.35) | 0.41 (0.03) |
| Adult | 26.13 (7.45) | 0.29 (0.04) | 0.74 (0.10) | 0.99 (0.12) | 0.35 (0.04) |
| **Epiphysis** | | | | | |
| Neonate | N/A | N/A | N/A | N/A | N/A |
| Infant 2 | N/A | N/A | N/A | N/A | N/A |
| Infant 2 | 27.18 (N/A) | 0.20 (N/A) | 0.56 (N/A) | 1.31 (N/A) | 0.30 (N/A) |
| Juvenile | 27.09 (15.61) | 0.30 (0.06) | 0.63 (0.13) | 1.11 (0.21) | 0.33 (0.09) |
| Adult | 13.20 (9.67) | 0.31 (0.05) | 0.71(0.09) | 0.99 (0.12) | 0.39 (0.08) |

**Figure S1.** Ontogenetic changes in BV/TV distribution from Neonate to Juvenile age categories in the *Gorilla* (species pooled) third metacarpal. Each specimen is shown in the midsagittal cross-section, including segmented trabecular and cortical bone (left) and the BV/TV distribution colour map scaled to 0.0-0.45 (right) (see Figure 6 for colour maps scaled to individual data range). Note specimen size not to scale.

**Figure S2.** *Gorilla* (species pooled) third metacarpal ontogenetic variation in BV/TV distribution within the Juvenile age category. Each specimen is shown in the midsagittal cross-section, including segmented trabecular and cortical bone (left), and the BV/TV distribution scaled from 0.0 – 0.45 (centre) and scaled to the range of each specimen (right).

**Figure S3.** Adult BV/TV distribution in the *Gorilla* (species pooled) third metacarpal. Each specimen is shown in the midsagittal cross-section of segmented trabecular and cortical bone and BV/TV distribution colour map scaled from 0.0 – 0.45. Note the occurrence of remnant epiphyseal line within the trabecular structure of most specimens (yellow arrow), including those of known older ages (e.g. GP 131 and GP 116). *G. g. gorilla* specimens are ordered in terms of degree of dental wear. Associated crania were not available to assess relative ages of ZMB 31624 and PC Mer 1.29. All specimens scaled to the same size.
