# Supplementary figures and images for: Ontogenetic changes to metacarpal trabecular bone structure in mountain and western lowland gorillas
Source: J Anat. 2022 Feb 4;241(1):82–100. doi: 10.1111/joa.13630 (PMC9178373; doi:10.1111/joa.13630)

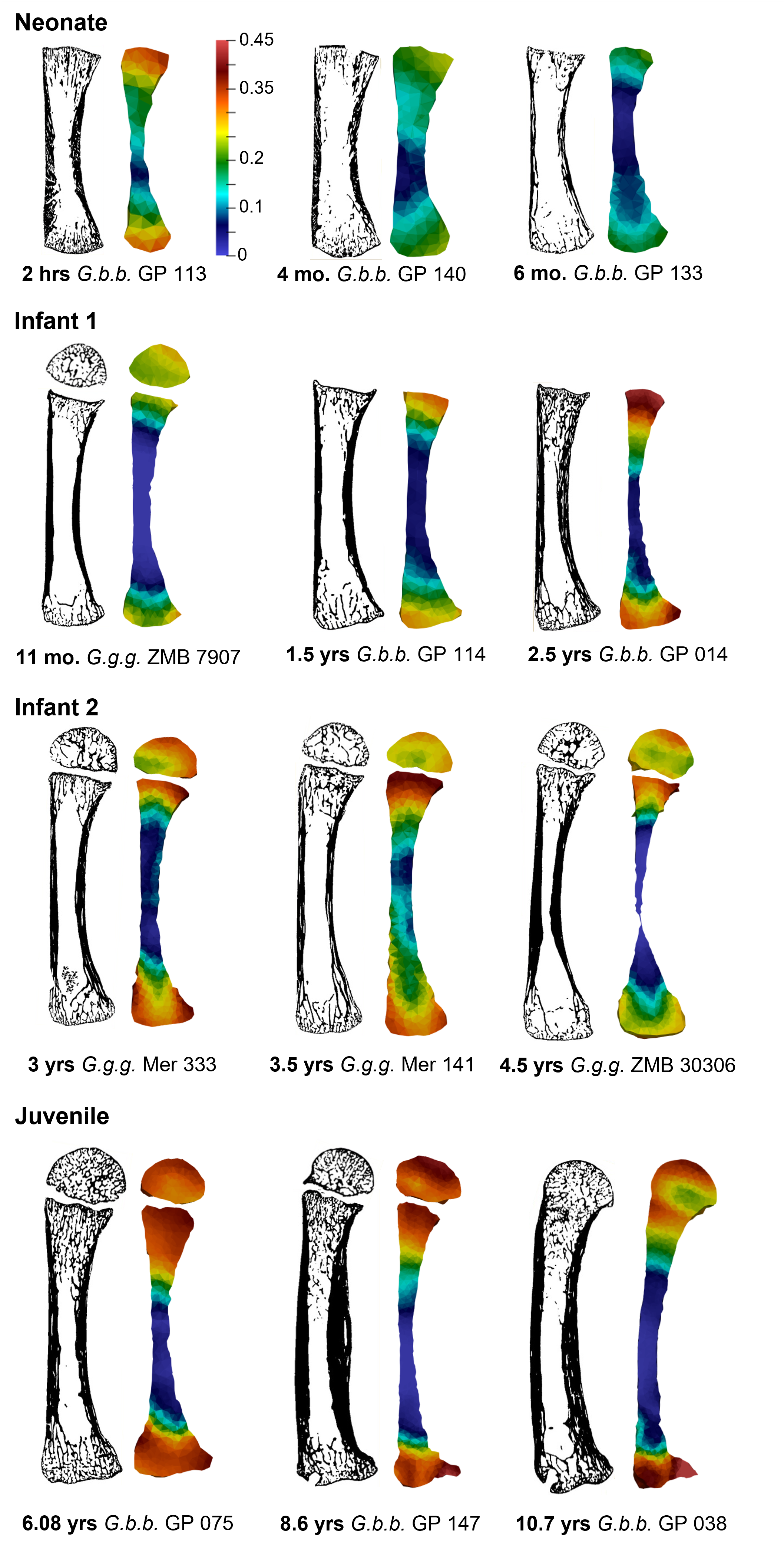

Supplement: Supplementary file 1 — Appendix S1 [file JOA-241-82-s001.zip › joa13630-sup-001-FigureS1.tif]

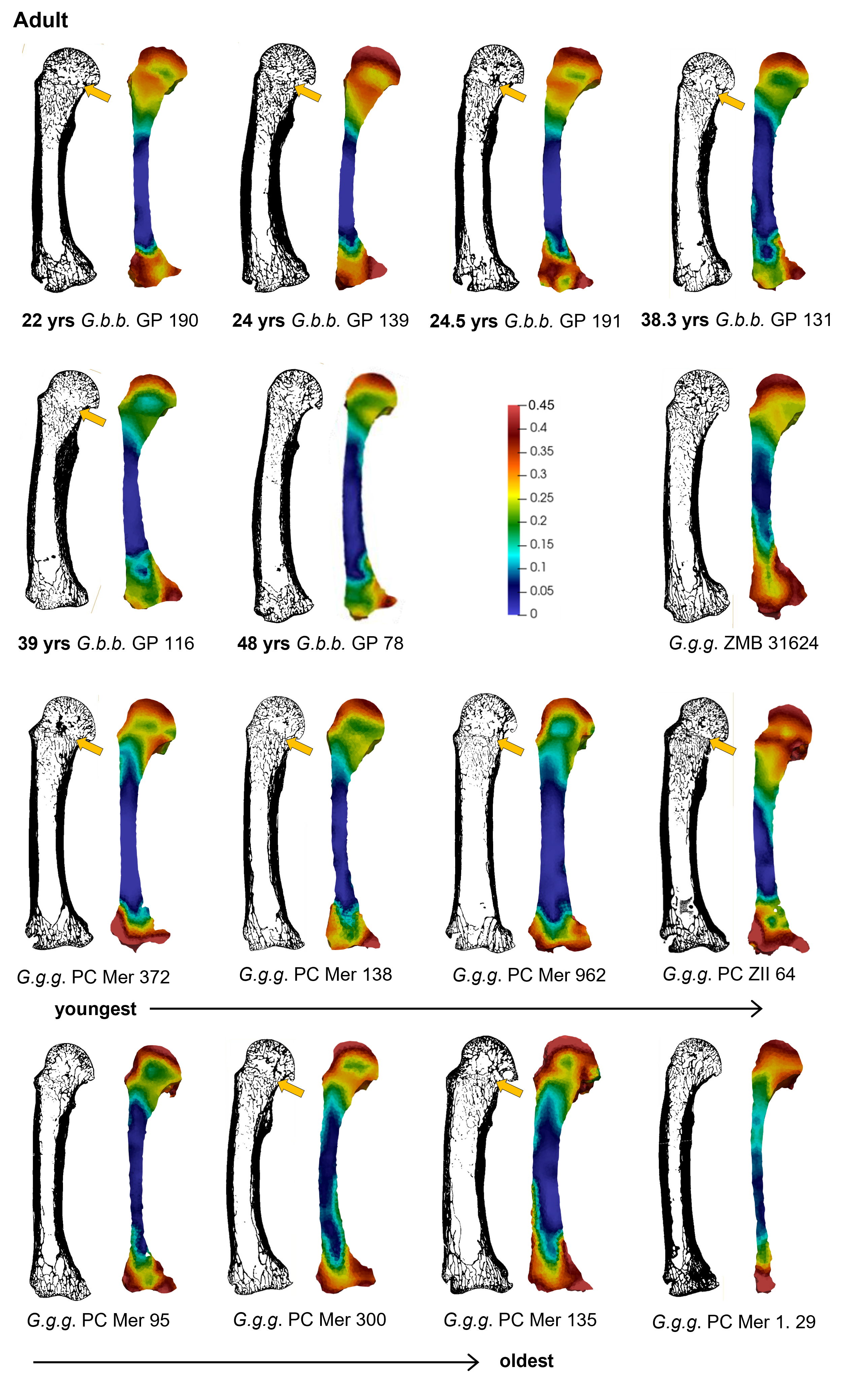

Supplement: Supplementary file 1 — Appendix S1 [file JOA-241-82-s001.zip › joa13630-sup-001-FigureS3.tif]

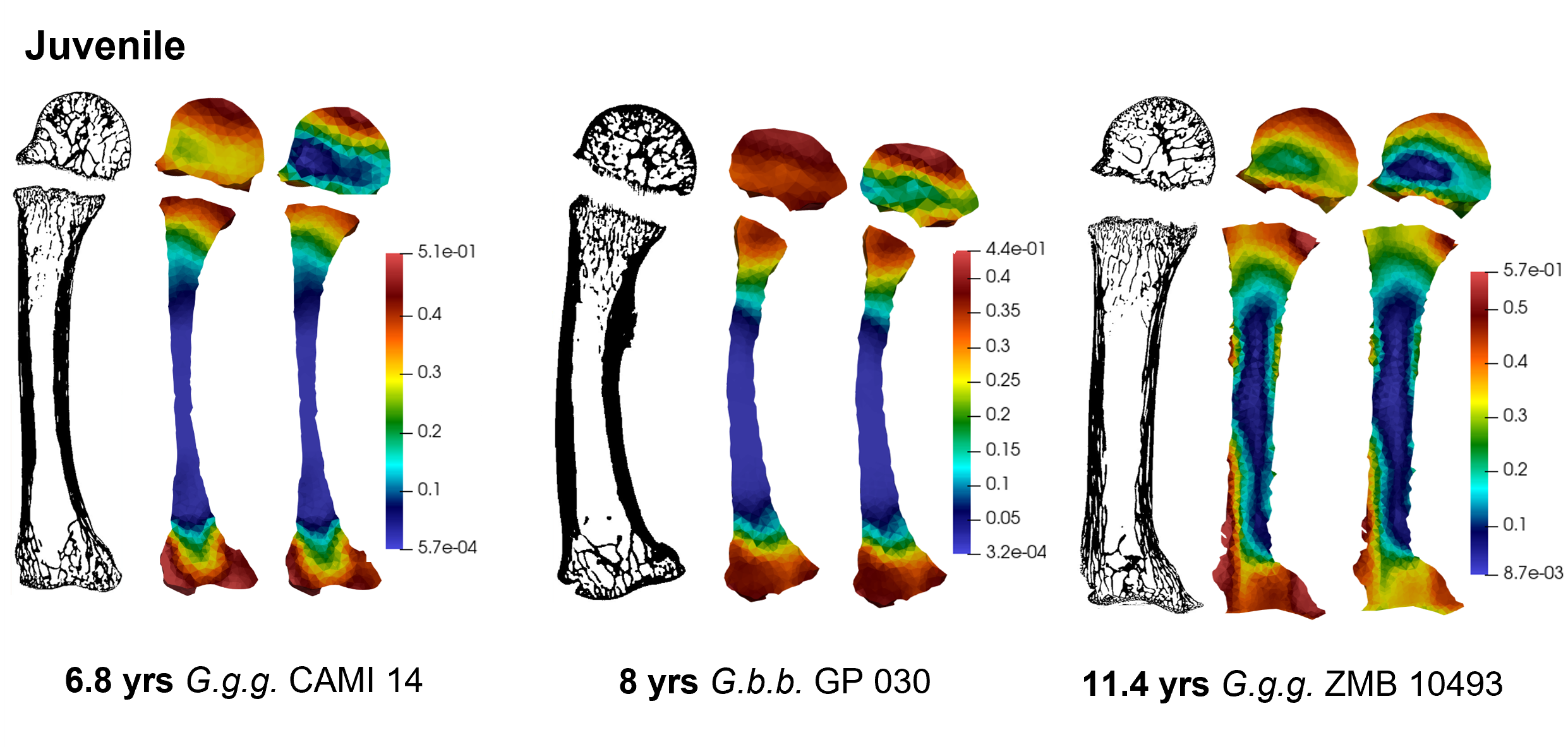

Supplement: Supplementary file 1 — Appendix S1 [file JOA-241-82-s001.zip › joa13630-sup-002-FigureS2.tif]
